# Pharmacokinetics of intramuscular alfaxalone and its echocardiographic, cardiopulmonary and sedative effects in healthy dogs

**DOI:** 10.1371/journal.pone.0204553

**Published:** 2018-09-24

**Authors:** Inga-Catalina Cruz-Benedetti, Isabelle Bublot, Thibault Ribas, Isabelle Fourel, Claus Vogl, Claire Dubois, Mathilde Milani, Keila Kazue Ida, Karine Portier

**Affiliations:** 1 Université de Lyon, VetAgro Sup, GREAT, Marcy l’Etoile, France; 2 Université de Lyon, VetAgro Sup, Unit of Cardiology, Marcy l’Etoile, France; 3 Azurvet, Hippodrome, Cagnes sur mer, France; 4 Université de Lyon, VetAgro Sup, INRA, USC1233, RS2GP, Marcy l’Etoile, France; 5 Department of Animal Husbandry and Genetics, Section for Molecular Biology, Vetmeduni, Vienna, Austria; 6 Laboratory of Medical Investigation 8, Anaesthesiology, Medical School, University of São Paulo, São Paulo, Brazil; 7 Univ Lyon, CarMeN Laboratory, INSERM, INRA, INSA Lyon, Université Claude Bernard Lyon 1, Bron, France; University of Bari, ITALY

## Abstract

The pharmacokinetics and the effects of a single intramuscular (IM) dose of alfaxalone on sedation and cardiopulmonary and echocardiographic variables was studied in dogs. Twelve healthy adult Beagles (3 females, 9 males) were used in this prospective controlled cross-over trial. Echocardiography was performed with and without 4 mg kg^-1^ alfaxalone IM with a week wash-out interval. Sedation (19-point scale; 0 = no sedation), cardiopulmonary parameters, blood gas analysis and plasma concentration of alfaxalone were assessed every 5 minutes following the injection (T0). The influence of the alfaxalone plasma concentration and time on physiological variables was tested using a linear model whereas echocardiographic measurements were compared between conscious and alfaxalone-administered dogs using paired t-tests. Compared to baseline, alfaxalone administration was followed by an increase in heart rate (HR) from T5 to T30 and a decrease in mean arterial pressure (MAP) at T10, T25 and T30, in stroke volume (SV; 15 ± 5 to 11 ± 3 ml; *P*<0.0001), and end-diastolic volume (EDV; 24.7 ± 5.7 to 19.4 ± 4.9 ml). Cardiac output (CO) and blood gas analysis did not change significantly throughout. Mean plasma half-life was 29 ± 8 minutes, volume of distribution was 1.94 ± 0.63 L kg^-1^, and plasma clearance was 47.7 ± 14.1 ml kg^-1^ minute^-1^. Moderate to deep sedation was observed from T5 to T35. Ten dogs showed paddling, trembling, nystagmus and strong reaction to sound during the procedure. Although there were no significant changes in CO and oxygenation, the impact of HR, MAP, SV, EDV alterations requires further investigations in dogs with cardiac disease.

## Introduction

Interpretation of echocardiography in canine patients requires high quality images, which can only be acquired in calm animals that do not resist physical restraint. In addition, patients presented for echocardiography may have severe cardiac diseases. A chemical restraint with minimal effect on blood pressure and cardiac output (CO) often becomes necessary to ensure myocardial oxygenation in these patients [1]. Ideally, interpretation of the echocardiographic variables should not be affected by sedation. However, most sedative and anaesthetic drugs (like alpha-2 agonists, acepromazine, ketamine) influence cardiac activity and vascular tone directly [2].

Alfaxalone in hydroxypropyl beta cyclodextrin, a neuroactive steroid anaesthetic, provides good short-term anaesthesia with minimal cardiorespiratory effect in unpremedicated dogs at 2–4 mg kg^-1^ IV [3–5]. According to Kim *et al*. [6], alfaxalone IV also provides an adequate sedation for echocardiographic examination with no statistical and clinical significant cardiovascular depression in healthy dogs. However, the IV administration route may not be feasible in aggressive, stressed or sick animals without previous sedation by the oral, intramuscular (IM) or subcutaneous route. In addition, IM injections can be less time-consuming and expensive than IV injections for which the placement of a catheter is recommended.

Moreover some authors suspected that the rapid alfaxalone IV injection is responsible for rare cardiorespiratory effects, particularly apnoea [5,7].

Alfaxalone’s hydrosolubility is increased by 375 times due to its excipient hydroxypropyl beta cyclodextrin [8], which makes its administration IM possible although this route is not approved in France. The choice of the administration route should be based on safety, efficacy, patient or clinician preference and pharmacoeconomics [9]. Alfaxalone administered IM induced dose-dependent neurodepression, lateral recumbency and maintenance of cardiovascular parameters within clinically acceptable limits [10]. Combinations of medetomidine and butorphanol administered as premedication prior to a subclinical dose of alfaxalone IM were not recommended in dogs with cardiovascular disease but the role of each drug remained unclear [11,12]. Alfaxalone in combination with butorphanol, provided a rapid, deep sedation in cats and induced some changes in echocardiographic measurements that were not considered clinically significant [13].

To our knowledge, the effect of IM alfaxalone alone on echocardiographic measurements in dogs has not been studied yet. Similarly, while the pharmacokinetics of IV alfaxalone administration in dogs have been investigated [14], such data following the IM route remained unknown. Therefore, the objective of our study was to determine the pharmacokinetics and dynamics of a single IM dose of alfaxalone and its impact on sedation, cardiopulmonary function, and echocardiographic measurements in healthy dogs. Deep sedation of approximately 30 minutes, an increase in heart rate (HR), a decrease in arterial blood pressure and respiratory rate (*f*R) with no clinically significant modifications in echocardiographic measures were expected.

## Materials and method

### Animals

The study was approved (N° 1453) by the Ethical Committee of VetAgro Sup (Chairperson Dr S. Vidal) on 15 December 2014. The study followed the recommendations of the Animal Research Reporting of In Vivo Experiments (ARRIVE) guidelines [15]. Twelve 2.0 ± 0.6 years old healthy Beagle dogs, 9 males and 3 females, weighing 9.3 ± 1.7 kg, with a body condition score of 4 out of 9 (range 3–7), and American Society of Anaesthesiologist physical status classification (ASA) 1–2 were included in the study. The sample size was determined by power analysis of 0.88 for one-sample experimental design to detect a difference in means of 30 beats minute^-1^ in HR, considering a standard deviation of 38 and a nominal significance level of 0.05. Dogs were judged to be healthy based on physical examination and on a complete blood count and serum biochemistry, including troponin I to rule out myocardial cell injury. The dogs were originated from a laboratory of our institution, where they were housed in groups with free access to an outdoor area. Their health status was monitored daily by a veterinarian (ICCB) and the study was performed in the Veterinary School of our institution.

### Study design

**Fig 1:**
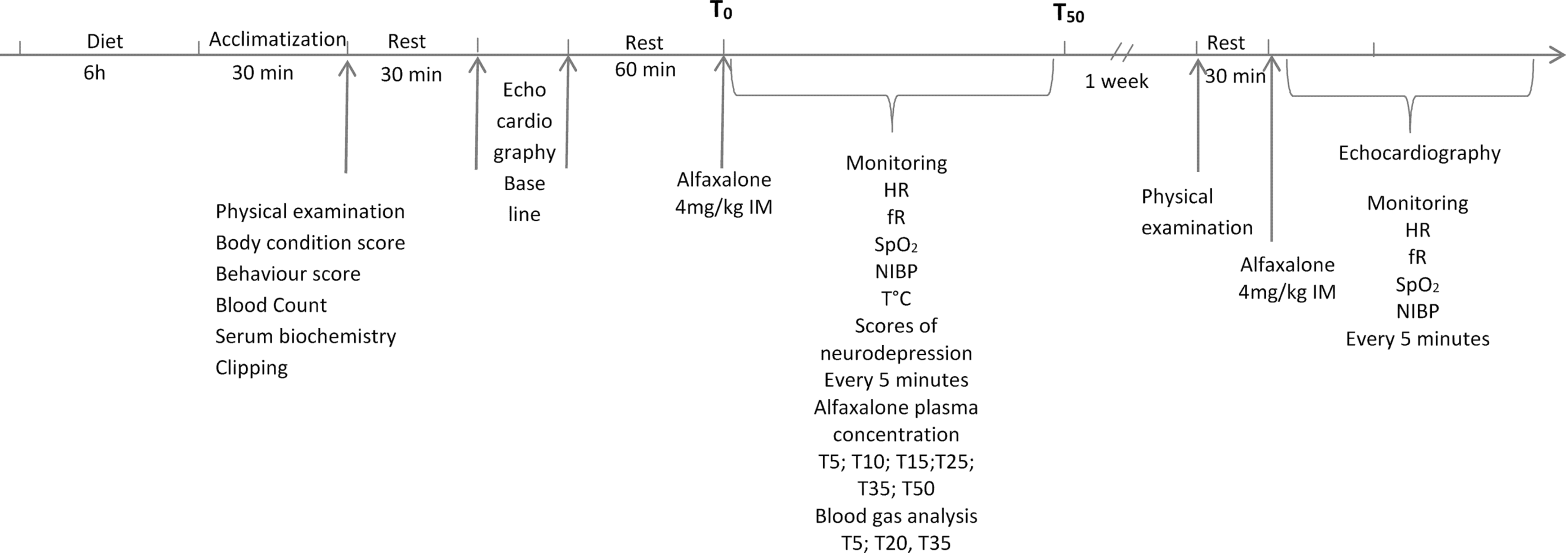
Study design.

In this prospective controlled cross-over experimental study the order in which animals underwent the study was randomly assigned by submitting the animal chip numbers to a web software (https://www.random.org/lists/) and attributing letters from A-L to the animals in the order assigned by the software. The study design is shown in Fig 1.

Dogs were fasted for 6 hours prior to be brought to the examination room. Each animal was given at least 30 minutes to adapt to the new environment before their behaviour was scored with a simple descriptive score (1 = calm, 2 = stressed/cooperative, 3 = stressed/aggressive, 4 = very aggressive). Baseline measurements of physiological variables were registered and a 4 ml venous blood sample was withdrawn from the jugular vein.

#### Echocardiography in conscious dogs

After a further resting time of at least 30 minutes, dogs were auscultated by a veterinary cardiologist (MM) and an echocardiographic assessment in lateral recumbency with a simultaneous lead II electrocardiogram (Vivid I, with 6S phased array probe, GE Healthcare, France) was performed in accordance with standard recommendations by another cardiologist (IB) to establish baseline values before sedation [16].

Echocardiographic data were recorded by a single blinded third cardiologist (TR) for off-line measurements. Measurements obtained from R-R interval over three consecutive cardiac cycles were averaged and compared to control values. Pulse rate was also registered independently every 5 minutes throughout. The echocardiography was always performed in the same order: 1) right parasternal window: right parasternal four-chamber long-axis view, right parasternal five-chamber long-axis view, right parasternal short-axis transventricular and transaortic view, and modified parasternal short-axis view, 2) subcostal view, 3) left parasternal window: left parasternal four-chamber long-axis view; left parasternal five-chamber long-axis view and a modified left parasternal short-axis view. The left ventricular posterior wall thickness (LVPW), interventricular septal wall thickness (IVS) and the left ventricular diameter (LVID) were measured using the M-Mode from a right parasternal transventricular short-axis view in diastole (d) and systole (s). The shortening fraction (FS) was calculated as a percentage according to the formula: FS = 100 × (LVIDd-LVIDs)/LVIDd. Left ventricular end-diastolic (EDV) and end-systolic volumes (ESV) were measured with single plane Simpson’s method 2D-mode still images from a right parasternal four-chamber long-axis view. The ejection fraction (EF) was calculated as a percentage from the formula: EF = 100 × (EDV-ESV)/EDV. The stroke volume (SV) was calculated as SV = EDV—ESV. The CO was calculated as CO = HR × SV. A right parasternal short-axis transaortic view was used to measure left atrial (LA) and aortic (Ao) diameters in early diastole from 2D still images [17] and the LA/Ao ratio was calculated. Peak pulmonic flow velocity (V_Pulm_max) was measured with continuous-wave Doppler from a modified right parasternal short-axis view as previously described [18]. Peak aortic flow velocity (V_Ao_ max) was measured with continuous-wave Doppler from a subcostal view. Isovolumic relaxation time (IVRT) was measured from a left parasternal five-chamber view. The peak velocity of the tricuspid regurgitation (TR) and the peak velocity of the pulmonary regurgitation were recorded by continuous-wave Doppler from any view that offered the highest velocity flow in order to evaluate systolic and/or diastolic pulmonary hypertension respectively. Mitral inflow was recorded by pulsed-wave Doppler from a left apical four-chamber view and used to estimate the peak velocity of early diastolic transmitral flow (E) and late diastolic transmitral flow (A) as well as to measure the E-wave deceleration (EDT) time. Mitral valve annulus velocities were measured from a left apical four-chamber view with Tissue Doppler Imaging (TDI). In order to measure the peak early diastolic lateral (E’_lat_), the peak early diastolic septal (E’_sept_), the peak late diastolic lateral (A’_lat_) and the peak late diastolic septal (A’_sept_) mitral valve annulus velocities the sample was placed successively on the septal and the lateral mitral valve annulus. The collected data were used to calculate the following ratios: E/A, E/E’_lat_, E/E’_sept_, E’_lat_/A’_lat_, E’_sept_/A’_sept_.

#### Sedation

After completion of the echocardiography, dogs were allowed to rest for an hour and were then injected 4 mg kg^-1^ alfaxalone (Alfaxan, 10 mg ml^-1^, Dechra-Jurox, UK) intramuscularly (IM). The drug was administered at two injection sites in the caudal lumbar muscle (T0), since the total volume exceeded 0.25 ml kg^-1^, which is considered good practice for injection in one site [19].

**Sedation score**

**Fig 2:**
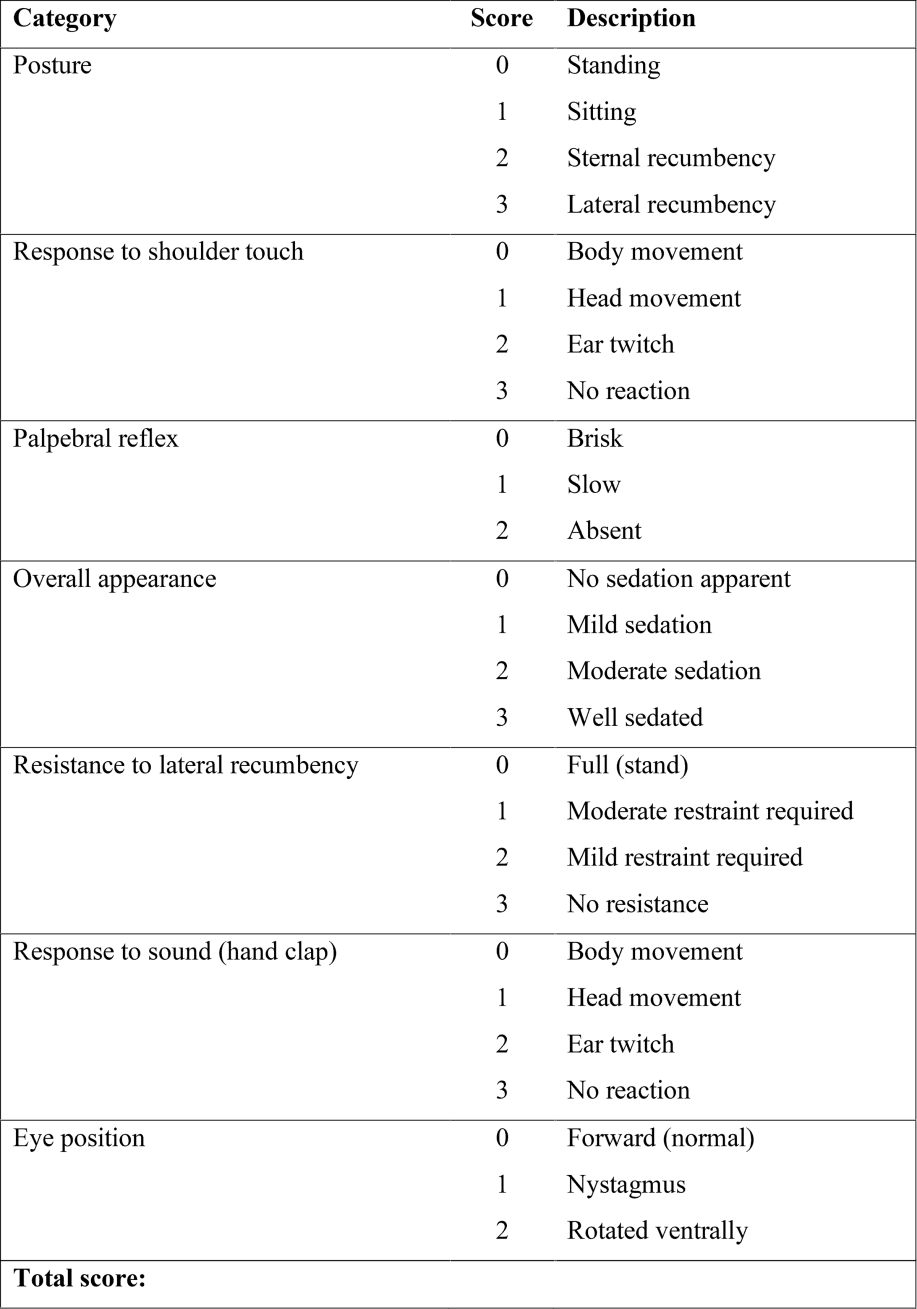
Modified scale of Gurney *et al*. [20] used to score sedation in 12 healthy Beagles every 5 minutes from 5 minutes after IM injection of 4 mg kg^-1^ alfaxalone until they were standing.

Sedation was video recorded and scored using a modified 19-points simple descriptive sedation scale [20] (0 = no sedation, 10–15 = moderate sedation, >15 = deep sedation; Fig 2) at baseline and every five minutes following alfaxalone injection until the animal stood up. The time to lateral recumbency (LRT), time to voluntary first movement (VFMT), time to head lift (HLT), time to unaided standing (UST), time from lateral recumbency to voluntary first movement (SedT), time from lateral recumbency to unaided standing (DecT) and time from head lift to unaided standing (RecT) were assessed. Any adverse behaviour, such as paddling, increased muscular tension and vocalisation were recorded.

**Cardiopulmonary data**

The HR, respiratory rate (*f*R), oxyhemoglobin saturation (SpO_2_), oscillometric non-invasive blood pressure (NIBP) and rectal temperature (T°C) were monitored using a pre-calibrated monitor (Vetcare, B. Braun, Germany) at baseline and every five minutes after alfaxalone injection until the dog stood up. Arterial blood gas analyses were performed at T+5, T+20, and T+35.

**Pharmacokinetics**

Venous blood (2.5 ml) was sampled at baseline and at 5, 10, 15, 25, 35 and 50 minutes (T+5, T+10, T+15, T+25, T+35 and T+50, respectively) after injection for measuring the alfaxalone plasma concentration. Blood was collected in heparinized tubes that were immediately placed on wet ice and were centrifuged (ca. 21°C, 2,500 cycles minute^-1^, 5 minutes) within 30 minutes. The plasma was harvested and stored at -70 to -80°C for later determination of alfaxalone concentration by protein precipitation [21] followed by liquid chromatography-mass spectrometry using an Agilent 1200 series HPLC and 6410B Triple Quadrupole (Agilent Technologies, Santa Clara, CA, USA). The latter was performed by homogenisation of 50 μL of plasma (defrosted at ambient temperature) with 200 μL of acetonitrile using a vortex for one minute followed by centrifugation for 10 minutes at 10,000 cycles minute^-1^. The supernatant was retrieved, filtered and then used for plasma concentration dosage. Plasma obtained from the blood sampled before sedation was used as a control group. This plasma was mixed with different quantities of alfaxalone (from 100–2500 ng ml^-1^) to create a calibration curve.

A mono-compartmental pharmacokinetic method was used to approximate the pharmacokinetic variables for each dog. A semi-logarithmic scale was used to plot the plasma concentration curve for alfaxalone versus time. A linear trend curve was drawn to extrapolate the plasma concentration time course from the measured concentration points and its slope was calculated (k_e_). The elimination half-life (t_1/2_) was calculated from the slope: t_1/2_ = ln2/k_e_. Alfaxalone plasma concentration at the time of administration (C_0_) was estimated from the linear trend curve and the plasma clearance (Clp) was calculated as follows: Clp = Dose/ C_0._ The volume of distribution (Vd) was calculated from the formula: Vd = Clp/k_e_.

#### Echocardiography under sedation

One week later, dogs were fasted for 6 hours. They were then allowed to acclimatise in the examination room for at least 30 minutes before they were administered 4 mg kg^-1^ of alfaxalone IM at two injection sites in the contralateral caudal lumbar muscle compared to the previous week. Five minutes after drug administration, the dogs were gently restrained for echocardiography. The same cardiologist (IB) performed the echocardiography of conscious and sedated dogs. The third cardiologist (TR) performed offline analysis. Heart rate, MAP, *f*R and SpO_2_ were also recorded.

### Statistical analysis

Semi-quantitative variables (i.e. body condition scores, behaviour scores, and sedation scores) and the duration of echocardiography (strongly dependent on animal behaviour) were treated non-parametrically and were reported as median (range). All other data were presented as mean ± SD since the Kolmogorov-Smirnov test indicated no significant deviation from a normal distribution. The plasma concentration measurements were log-transformed after adding 10 to avoid undetermined numbers, i.e., *log*([alfaxalone]+10). The Bonferroni correction and the calculation of a local false discovery rate (FDR; q-value) were performed to account for multiple testing. Using a linear model, the influence of the alfaxalone plasma concentration was tested on six target variables (HR, MAP, *f*R, SpO_2_, T°C and sedation scores). A one-way repeated measures ANOVA followed by t-tests (alpha = 0.05/6 = 0.008) was used to detect differences between the baseline values of these variables and their values recorded at each of the six treatment times (T+5 to T+30). Echocardiographic measurements (24 target variables, where ratios were excluded as composites) were compared between conscious and alfaxalone-administered dogs using paired t-tests (alpha = 0.05/24 = 0.002); for these variables, a local FDR was also calculated at a level of 0.01 to compare to the results with the Bonferroni correction.

## Results

Data from one dog had to be excluded from the sedation trial since it moved during injection and the drug was partially administered subcutaneously.

**Fig 3:**
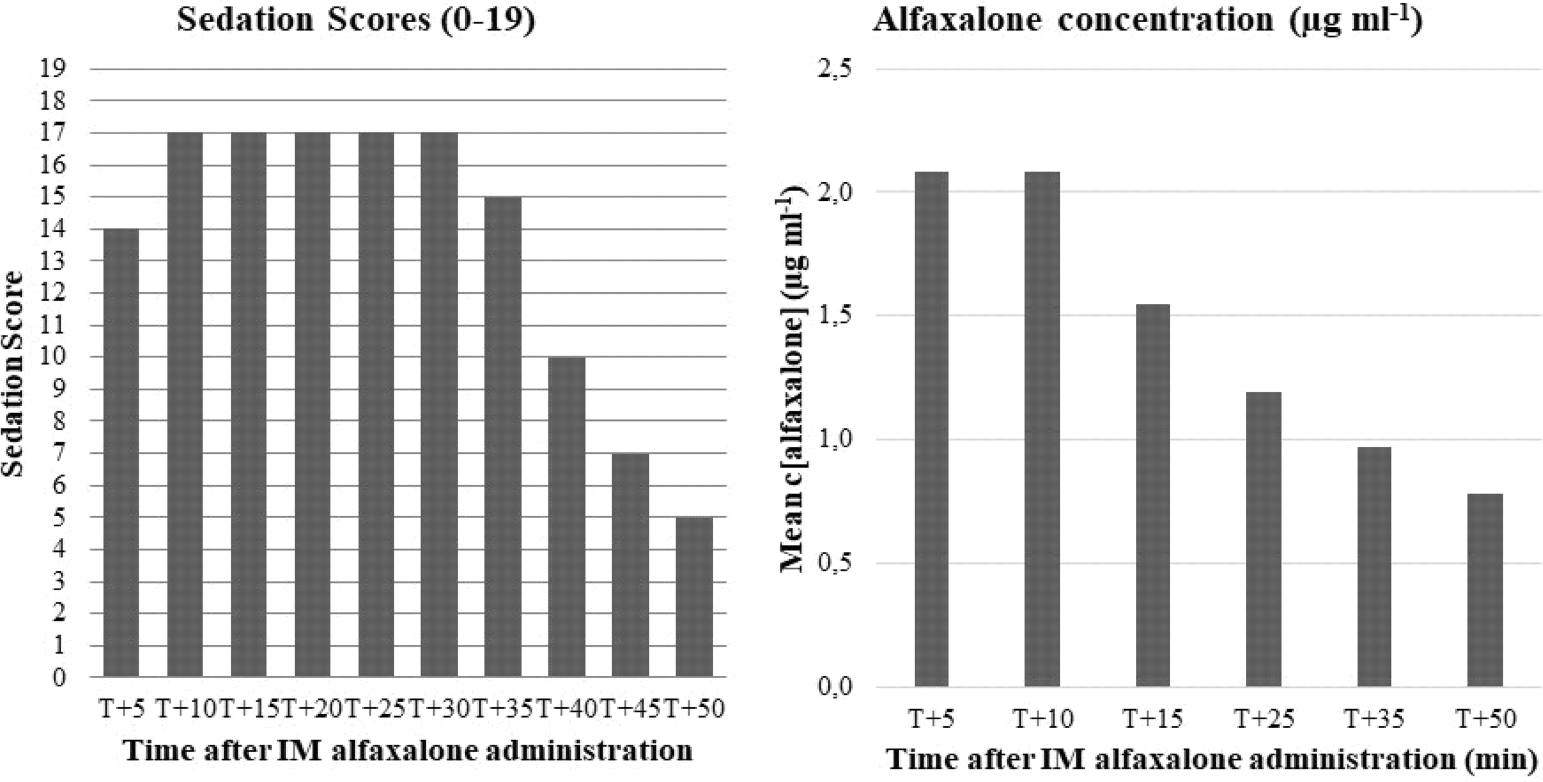
Median sedation scores and corresponding mean alfaxalone plasma concentration assessed every five minutes after IM administration of 4 mg kg^-1^ of alfaxalone in 11 healthy Beagles.

Results of alfaxalone plasma concentration are shown in Table 1 and Fig 3. The mean t_1/2_ was 29 ± 8 minutes, the Vd was 1.94 ± 0.63 L kg^-1^ and the Clp of alfaxalone was 47.7 ± 14.1 mL kg^-1^ minute^-1^.

**Table 1:** Mean (± SD) cardiorespiratory variables, alfaxalone plasma concentration, and median (range) sedation scores following 4 mg kg^-1^ alfaxalone administered intramuscularly in healthy Beagles during sedation alone (week I) and during echocardiography under sedation (week II).

**Week I**	Variables	Baseline	T+5	T+10	T+15	T+20	T+25	T+30	T+35	T+40	T+45	T+50
	HR (beats minute^-1^)	91 ± 11	119 ± 24*	138 ± 27*	135 ± 32*	137 ± 37*	122 ± 28*	122 ± 40*	-	-	-	-
	MAP (mmHg)	104 ± 14	102 ± 17	88 ± 11*	93 ± 23	92 ± 9	88 ± 12*	88 ± 11*	-	-	-	-
	*f*R (breaths minute^-1^)	26 ± 7	21 ± 7*	21 ± 6	22 ± 10	25 ± 13	19 ± 5	23 ± 12	-	-	-	-
	SpO_2_ (%)	NA	96 ± 2	96 ± 2	95 ± 4	96 ± 2	96 ± 3	96 ± 3	-	-	-	-
	T°C	38.4 ± 0.3	38.1 ± 0.4*	37.7 ± 0.3*	37.7 ± 0.4*	37.5 ± 0.4*	37.5 ± 0.4*	37.4 ± 0.5*	-	-	-	-
	Alfaxalone plasma concentration (μg ml^-1^)	0.0± 0.0	2.1 ± 0.6	2.1 ± 0.9	1.5 ± 0.4	-	1.2 ± 0.3	-	1.0 ± 0.3	-	-	0.8 ± 0.2
	Sedation score	0 [0;1]	14 [3;17]	17 [8;18]	17 [9;18]	17 [6;18]	17 [7;18]	17 [8;19]	15 [5;18]	10 [2;18]	7 [0;15]	5 [0;11]
**Week II**	HR (beats minute^-1^)	97 ± 13	114 ± 27	126 ± 22*	125 ± 24*	121 ± 21*	119 ± 29	104 ± 13	-	-	-	-
	MAP (mmHg)	100 ± 14	87 ± 12*	84 ± 12*	86 ±16*	83 ±11*	87 ± 14*	81 ±9*	-	-	-	-
	*f*R (breaths minute^-1^)	23 ± 4	18 ± 3*	18 ± 3*	18 ± 4*	18 ± 5	18 ± 3	19 ± 3	-	-	-	-
	SpO_2_ (%)	NA	96 ± 1	96 ± 1	96 ± 2	96 ± 2	97 ± 2	98 ± 1	-	-	-	-

HR: heart rate; MAP: mean arterial pressure; *f*R: respiratory rate; SpO_2_: oxyhemoglobin saturation; baseline: prior to alfaxalone administration; T+5, T+10, T+15, T+20, T+25, T+30, T+35, T+40, T+45 and T+50: 5, 10, 15, 20, 25, 30, 35, 40, 45 and 50 minutes after alfaxalone administration, respectively

* indicates a significant difference from baseline values after Bonferroni correction (*p* < 0.05/6 = 0.0083).

**Fig 4:**
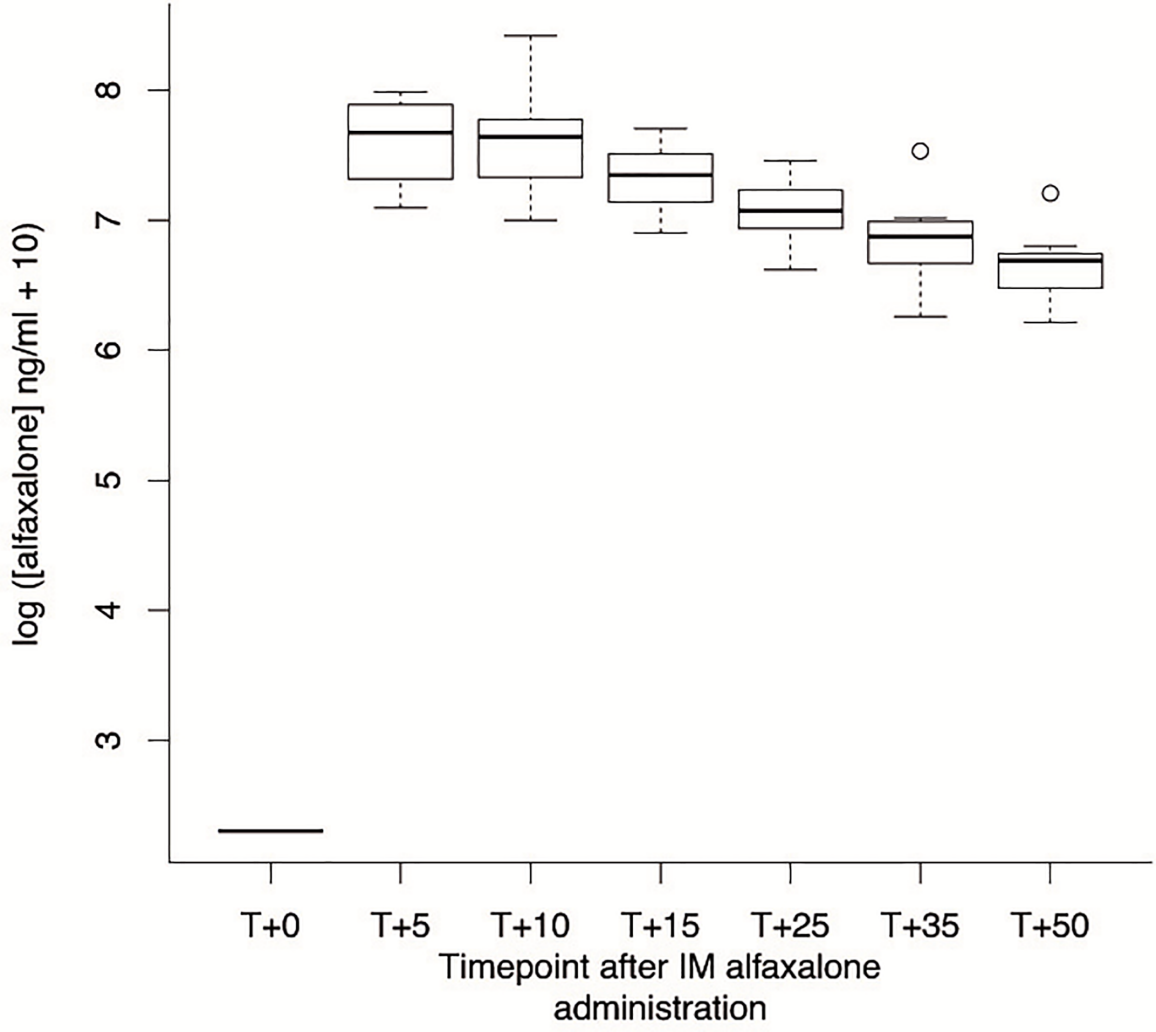
Boxplot of the log of the plasma concentration of alfaxalone at different time-points after application. Note the nearly linear relationship from T+10 onwards. T+0: alfaxalone administration; T+5, T+10, T+15, T+25, T+35, and T+50: 5, 10, 15, 25, 35, and 50 minutes after alfaxalone administration.

A nearly linear relationship between the logarithm of alfaxalone plasma concentration and time was observed from T+10 onwards (Fig 4). Alfaxalone plasma concentration influenced HR (regression coefficient = 7.43, *p* < 0.001) and the sedation score (regression coefficient = 2.810, *p* < 0.001) positively, and *f*R (regression coefficient = -1.16, *p* < 0.005) and T°C (regression coefficient = -0.13, *p* < 0.001) negatively. Alfaxalone plasma concentration had no significant influence on SpO_2_ (*p* = 0.97) and MAP (regression coefficient = -2.22, *p* = 0.03) after multiple test correction.

Under sedation alone (week I), a significant increase in HR was observed at T+5 (*p* = 0.003), T+10 to T+30 (*p* < 0.001); a significant decrease in MAP was observed at T+10 (*p* = 0.005), T+25 (*p* = 0.007), and T+30 (*p* = 0.006); a significant decrease in *f*R was observed at T+5 (*p* = 0.005); and a significant decrease in temperature was observed at all time points, T+5 (*p* = 0.004), T+10 to T+30 (*p* < 0.001). The SpO_2_ and arterial blood gas values did not change significantly throughout (Tables 1 and 2). Under sedation during echocardiography (week II), a significant increase in HR was observed at T+10 (*p* = 0.001), T+15 (*p* = 0.001), and T+20 (*p* = 0.005); a significant decrease in MAP was observed at T+5 (*p* = 0.002), T+10 to T+20 (*p* < 0.001), T+25 (*p* = 0.001), and T+30 (*p* = 0.002); a significant decrease in *f*R was observed at T+5 (*p* = 0.006), T+10 (*p* = 0.006), and T+15 (*p* = 0.008) (Table 1). The HR, MAP and *f*R acquired on alfaxalone-administered dogs in week I and II showed a similar trend, although all values were lower during the second sedation.

**Table 2:** Mean (± SD) arterial blood gas analysis following 4 mg kg^-1^ alfaxalone administered intramuscularly in 11 healthy Beagles.

Variables	T+5	T+20	T+35
pH	7.43 ± 0.03	7.43 ± 0.03	7.42 ± 0.03
HCO_3_^-^ (mmol/L)	22.2 ± 3.3	22.9 ± 3.5	23.0± 3.5
PaCO_2_ (mmHg)	36.5 ± 4.4	37.3 ± 4.9	38.5 ± 4.9
Anion Gap (mmol/L)	22.8 ± 2.1	22.9 ± 2.4	23.0 ± 2.3
BE (mmol/L)	-0.9 ± 2.8	-0.3 ± 3.0	-0.6 ± 3.0
tCO_2_ (mmol/L)	23.3 ± 3.5	24.1 ± 3.6	24.2 ± 3.6
PaO_2_ (mmHg)	85.0 ± 15.1	91.8 ± 9.4	88.3 ± 13
tHb (g/dL)	13.0 ± 2.0	12.0 ± 2.7	12.7 ± 2.3
SaO_2_ (%)	95.5 ± 2.9	95.8 ± 3.1	96.2 ± 3.4
Na^+^ (mmol/L)	156.4 ± 2.1	157.6 ± 2.0	158.1 ± 2.5
K^+^ (mmol/L)	3.3 ± 0.5	3.4 ± 0.5	3.2 ± 0.5
Cl^-^ (mmol/L)	114.7 ± 1.3	114.6 ± 2.5	115.2 ± 1.3
P_A_O_2_ (mmHg)	97.9 ± 3.9	97.0 ± 5.2	95.5 ± 4.7

HCO_3_^-^: plasma bicarbonate concentration; PaCO_2_: partial pressure of carbon dioxide in arterial blood; BE: base excess; tCO_2_: total carbon dioxide concentration; PaO_2_: partial pressure of oxygen in arterial blood; tHb: total haemoglobin concentration; SaO_2_: arterial oxygen saturation; Na^+^: sodium plasma concentration; K^+^: potassium plasma concentration; Cl^-^: chloride plasma concentration; PAO_2_: alveolar partial pressure of oxygen (= PaO_2_ x (P_atm_-47)-1.2 x PaCO_2_); T+5, T+20, and T+35: 5, 10, and 35 minutes after alfaxalone administration, respectively.

The median behavioural score was 1 (1–3). All dogs but one had behaviour scores of 2 or less and only one dog (J) was aggressive. The dogs were moderately to deeply sedated from T+5 to T+35. Sedation was considered deep for 20 minutes from T+10 onwards (Table 1). While the plasma concentration of alfaxalone was maximal at T+5, the sedation score had not reached its highest value (Fig 3). Conversely, the plasma concentration of alfaxalone started to decrease at T+15 while the sedation score was always maximal. There was a 20-minute lag between the decrease in the sedation score and the decrease in plasma concentration. Trembling or paddling after stimulation, almost exclusively by sound, were noted in 10 dogs (91%). Three of these dogs presented nystagmus during sedation.

The LRT was 5.4 ± 3.6 minutes, VFMT was 28.9 ± 10.0 minutes, HLT was 31.9 ± 8.0 minutes, UST was 48.2 ± 11.7 minutes, SedT was 23.5 ± 12.2 minutes, DecT was 42.8 ±14.1 minutes, and RecT was 16.3 ± 8.2 minutes.

### Echocardiography

Median duration of echocardiography in conscious and alfaxalone-administered dogs was 37 [22–60] minutes and 27 [24–33] minutes, respectively. All echocardiographic results are displayed in Table 3. Concerning the 2D echocardiographic measurements, EDV (*p* < 0.001) and SV (*p* < 0.001) were significantly lower in dogs administered alfaxalone compared to conscious dogs. Alfaxalone had no effect on CO, ESV, LA, LA/Ao, Ao, and EF. Regarding the M-mode echocardiographic measurements, no significant difference was observed in LVIDd, LVIDs, LVPWd, LVPWs, IVSd, IVSs or in FS. Concerning the Doppler echocardiographic measurements, significant decreases in E’_sept_ and V_Ao_ max (*p* < 0.001 for both variables) were observed between conscious and alfaxalone-administered dogs. No significant difference was observed in E velocity, E/A, EDT, V_pulm_max, A velocity, E’sept/A’sept, IVRT and E/IVRT. A local false discovery rate was also calculated using 24 variables (excluding the ratios as composite variables) with a cut-off of 0.01 and obtained the same three variables (EDV, E’sept, and V_Ao_ max) that were significantly different with the Bonferroni correction.

**Table 3:** Mean (± SD) echocardiographic measurements in 12 healthy conscious and alfaxalone-administered (4 mg kg^-1^ intramuscularly) Beagles.

Variables	Baseline	Sedation	*p*-value
	2D Measurements		
CO (L/min)	1.5 ± 0.4	1.4 ± 0.4	*p* = 0.29
SV (ml)	15 ± 5	11 ± 3*	*p* < 0.001
EDV (ml)	24.7 ± 5.7	19.4 ± 4.9*	*p* < 0.001
ESV (ml)	9.4 ± 1.8	8.5 ± 2.3	*p* = 0.1
LA diameter (mm)	24.1 ± 2.9	21.1 ± 2.4	*p* = 0.04
Ao diameter (mm)	18.1 ± 1.7	17.9 ± 1.4	*p* = 0.67
LA/Ao	1.33 ± 0.11	1.18 ± 0.1	*p* = 0.01
EF (%)	69.9 ± 7.2	56.3 ± 6.3	*p* = 0.18
	M-Mode echocardiographic		
LVIDd (mm)	29.2 ± 2.9	28.2 ± 3.0	*p* = 0.29
IVSd (mm)	7.5 ± 0.9	7.5 ± 1.6	*p* = 0.9823
LVPWd (mm)	7.5 ± 1.2	8.0 ± 1.3	*p* = 0.46
LVIDs (mm)	19.9 ± 3.3	18.7 ± 1.9	*p* = 0.25
IVSs (mm)	10.3 ± 1.5	10.0 ± 1.6	*p* = 0.52
LVPWs (mm)	11.0 ± 1.9	9.9 ± 1.2	*p* = 0.1
FS (%)	31.9 ± 6.5	33.7 ± 4.3	*p* = 0.52
	Doppler echocardiographic measurements		
E velocity (m s^-1^)	0.62 ± 0.09	0.51 ± 0.08	*p* = 0.007
A velocity (m s^-1^)	0.38 ± 0.10	0.40 ± 0.14	*p* = 0.27
E/A	1.75 ± 0.46	1.37 ± 0.37	*p* = 0.009
EDT (ms)	130.8 ± 27.5	93.0 ± 21.4	*p* = 0.005
E’lat (ms)	0.14 ± 0.08	0.08 ± 0.02	*p* = 0.02
A’lat (ms)	0.11 ± 0.09	0.05 ± 0.02	*p* = 0.04
E’lat/A’lat	1.42 ± 0.52	1.55 ± 0.64	*p* = 0.44
E’sept (ms)	0.07 ± 0.01	0.05 ± 0.01*	*p* < 0.001
A’sept (ms)	0.05 ± 0.01	0.05 ± 0.02	*p* = 0.1
E’sept/A’sept	1.40 ± 0.25	1.18 ± 0.41	*p* = 0.13
E/E’lat	5.28 ± 1.71	7.27 ± 1.81	*p* = 0.004
E/E’sept	8.75 ± 2.18	11.10 ± 2.32	*p* = 0.02
IVRT (ms)	74.7 ± 20.4	86.6 ± 12.2	*p* = 0.18
E/IVRT	0.010 ± 0.007	0.006 ± 0.002	*p* = 0.1
V_Ao_ max (m s^-1^)	1.22 ± 0.10	1.01 ± 0.10*	*p* < 0.001
V_pulm_max (m s^-1^)	1.1 ± 0.17	0.93 ± 0.20	*p* = 0.01
	Valvular insufficiency		
TR	1.97 ± 0.43	1.96 ± 0.33	*p* = 0.8
PR	0.90 ± 0.17	1.36 ± 0.30	*p* = 0.01

CO: cardiac output; SV: stroke volume; EDV: left ventricular end-diastolic volume; ESV: left ventricular end-systolic volume; LA: left atrial diameter in early diastole; Ao: aortic diameter in early diastole; LA/Ao: left atrial diameter to aortic diameter ratio; EF: ejection fraction; LVIDd: left ventricular internal dimension in diastole; IVSd: interventricular septum in diastole; LVPWd: left ventricular posterior wall in diastole; LVIDs; left ventricular internal dimension in systole; IVSs interventricular septum in systole; LVPWs: left ventricular posterior wall in systole; FS: fractional shortening; E velocity: peak velocity of early diastolic transmitral flow; A velocity: peak velocity of late diastolic transmitral flow; EDT: E-wave deceleration; E’ lat: peak early diastolic lateral mitral valve annulus velocity; A’ lat: peak late diastolic lateral mitral valve annulus velocity; E’ sept: peak early diastolic septal mitral valve annulus velocity; A’ sept: peak late diastolic septal mitral valve annulus velocity; IVRT: isovolumetric relaxation time; V_Ao_ max: peak aortic flow velocity; V_Pulm_max: peak pulmonic flow velocity.

*values are different from Baseline with *p* < 0.002 (Bonferroni correction: 0.05/24 = 0.002). A local false discovery rate was calculated using 24 variables (excluding the ratios as composite variables) with a cut-off of 0.01 and obtained the same three variables (EDV, E’sept, and V_Ao_ max) that were significantly different with the Bonferroni correction.

Mild tricuspid regurgitation was present in all dogs. The peak velocity of the TR could be measured in all dogs but one after sedation because the flow profile was poorly defined in that dog. Mild pulmonary regurgitation was present in 6 dogs at baseline and in 8 dogs after sedation. None of the recorded maximum velocities were indicative of systolic and/or diastolic pulmonary hypertension according to previously published data (> 2.80 m/sec for tricuspid regurgitation and 2.20 m/sec for pulmonary regurgitation) [22].

### Adverse effects

Paddling, trembling and nystagmus were noted in 6 alfaxalone-administered dogs (50%) during echocardiography at T+20 (A), at T+2 and T+31 (E), at T+15 and T+17 (G), at T+32 (H), at T+4, T+22, and T+24 (L), and at T+19 and T+21 (K). External noises excited two of these dogs (G and L) and the echocardiographic examination had to be paused for a few minutes (at T+19 and T+24, respectively) in order for them to calm down.

## Discussion

To our knowledge, this is the first report on the pharmacokinetics and echocardiographic effect of IM alfaxalone alone in dogs. In the present study, the IM administration of 4 mg kg^-1^ of alfaxalone to healthy Beagles induced moderate to deep sedation for 30 minutes and was associated with a significant increase in HR and a significant decrease in SV, EDV, MAP, *f*R, and rectal temperature. The CO and systemic oxygenation remained unchanged after IM alfaxalone. Adverse reactions such as an increased reactivity to sound, trembling, paddling and nystagmus were observed during sedation.

Several studies report the cardiovascular effects of alfaxalone administered IV alone in healthy dogs. They differ in the population, the route of administration, the dose, the type of qualitative scores used (to assess the quality of induction, neurodepression and recovery) and the definition of the different times studied (sedation, recovery). However, despite these differences most of them seem to agree with our findings, indicating that a dose of alfaxalone greater than 2 mg kg^-1^ IV induces a dose-dependent increase in HR and a decrease in blood pressure [3–6].

The tachycardia can be a limiting factor for the use of alfaxalone during echocardiography. Some authors highlighted that alfaxalone might be contraindicated in patients in whom an excessive rise in HR could increase the risk of myocardial ischaemia. In addition, an increase in HR may interfere with acquisition of high quality echocardiographic images. Slow IV administration was suggested to reduce the short-lived alfaxalone-induced tachycardia [5]. Tamura *et al*.’s studies [10,12] demonstrated that the haemodynamic effects may be more moderate with no increase in HR when alfaxalone at doses of 2.5, 5, 7.5 and 10 mg kg^-1^ was administered IM. These findings suggested that this administration route might have less deleterious effect on the cardiovascular function than the IV route, although this was not confirmed by our study. Unfortunately, in Tamura *et al*.*’s* studies, the population was small with a large distribution of age and body weight, which may not have achieved sufficient statistical power.

In our study, the increase in HR could be associated with the decrease in blood pressure through the baroreceptor reflex. A decrease in systemic vascular resistance was more likely to be the mechanism of blood pressure reduction, since CO was maintained by compensatory responses between HR increase and SV decrease. This was supported by previous studies that observed an association of IV-alfaxalone-induced tachycardia with either no change in CO [4] or an increase in cardiac index [5] and a decrease in systemic vascular resistance [4,5]. Additionally, vasodilatation may have been responsible for the continuous decrease in body temperature, which was also reported in other studies [4,12], and the use of heating devices during alfaxalone sedation may be advisable.

The present study identified only minor changes in echocardiographic variables following IM alfaxalone alone in healthy dogs. The decrease in EDV and E’sept was most likely a direct consequence of the increased HR, due to the decreased time for ventricular filling during diastole. The decrease in V_Ao_ max might have been a consequence of the decrease in EDV. However, the magnitude of changes in these variables was low and most likely of no clinical relevant. Accordingly, the IV administration of 3 mg kg^-1^ alfaxalone in healthy dogs also showed only clinically insignificant variations in echocardiographic variables and HR [6]. To our knowledge there are no studies characterizing the echocardiographic effects of an IM injection of alfaxalone alone in dogs. Seo *et al*. [23] studied the combination of 2 mg kg^-1^ alfaxalone IM with butorphanol and midazolam and showed a non-statistically significant reduction on echocardiographic left ventricular indices. Lee *et al*. [11] observed a decrease in HR, CO, SV, LVEF and FS and an increase in LVIDs following the combination of 2 mg kg^-1^ alfaxalone IM with butorphanol and medetomidine but the role of each drug on myocardial depression remained unclear. In cats, the association of butorphanol (0.2 mg kg^-1^) and alfaxalone (2 mg kg^-1^) IM induced only a mild decrease in HR. The effects on echocardiographic measurements were not considered clinically relevant except for the reduction in FS and EF, which are markers of left ventricular function [13]. Studies specifically designed to look at the echocardiographic effects of this drug combination in dogs are lacking and might be of high clinical interest.

In the present study, *f*R decreased significantly during both simple sedation and sedation for echocardiography. These results are in accordance with Tamura *et al*.’s study [10] who found a significant decrease in *f*R in dogs after IM alfaxalone administered at 5 mg kg^-1^, 7.5 mg kg^-1^, and 10 mg kg^-1^. Muir *et al*. [4] also found a dose-dependent decrease in *f*R and minute volume immediately after induction with IV alfaxalone at 2 mg kg^-1^, 6 mg kg^-1^ and 10 mg kg^-1^ in dogs. Episodes of apnoea and hypoxemia were observed following the highest doses. In our study, as well as in Tamura *et al*.’s study, apnoea was not observed and the decrease in *f*R had no effect on arterial blood gas analysis, suggesting it might have been clinically non-significant. The IV route might induce more severe respiratory depression than the IM route. Decreased *f*R was also observed when alfaxalone was used in combination with other sedative drugs [11, 24].

Body temperature decreased slightly with time (loss of 1°C), which was not surprising during anaesthesia. The temperature was negatively associated with the alfaxalone plasma concentration, despite the latter also decreasing with time. These data seemed conflicting but could be explained if we consider the observation times and duration of the study. The alfaxalone plasma concentration decreased with time only from T15. However, the greatest temperature reduction, which was accompanied by the maximal increase in alfaxalone concentration, was observed within the first 10 minutes after drug administration. The decrease in systemic vascular resistance induced by alfaxalone could explain the heat loss, which was certainly more important during the first part of the sedation.

The mean t_1/2_ (29 ± 8 minutes), Vd (1.94 ± 0.63 L kg^-1^) and Clp (47.7 ± 14.1 mL kg^-1^ minute^-1^) were similar to those published by Ferré *et al*. [13] in Beagles after IV administration of half of our dose of alfaxalone (2 mg kg^-1^), i.e. 24.0 ± 1.9 minutes, 2.4 ± 0.9 L kg^-1^, and 59.4 ± 12.9 mL kg^-1^ minute^-1^, respectively. Pasloske *et al*. [25] also found similar values in unpremedicated Greyhounds that were administered 2 mg kg^-1^ alfaxalone IV. These values indicated a relatively rapid elimination of the molecule and good diffusion in the organism (large volume of distribution). Ferré *et al*. [14] detected maximum plasma concentrations at 2 minutes after IV injection of 2 mg kg^-1^ alfaxalone. In our study, the first sampling was withdrawn at 5 minutes after IM administration to avoid the influence of external stress and to allow for the animal to become sedated and recumbent. Venous blood samples prior to 5 minutes after injection would have been necessary to determine the maximum plasma concentration after IM administration. This would have improved the precision of pharmacokinetic data. Furthermore, additional alfaxalone plasma concentration analysis at least every hour for three hours, might have allowed to record the time needed to clear the plasma from the drug.

In our study, we wanted to characterize the effects of IM alfaxalone alone on cardiovascular function. However other authors have suggested that the combination of alfaxalone IM with other molecules could have many advantages in premedication [11, 12]. The synergic sedative effect with other drugs produces sparing effects on the dose of IM alfaxalone and reduces the large volume required to produce clinical sedation compared to alfaxalone alone. It is also expected that the combination provides analgesia and better quality of anaesthesia and recovery. However, some combinations, such as with medetomidine, were not advisable for patients with cardiac disease as the beneficial sedative effects are overcome by a significant bradycardia most likely caused by a baroreceptor mediated reflex associated with the alpha-2 adrenoceptor agonist [12]. Alfaxalone associated with butorphanol produced good and safe sedation for echocardiography in cats [13] and, therefore, it might improve the quality of sedation in dogs without challenging the cardiovascular function.

Based on the sedation scores dogs were moderately to deeply sedated for a period of approximately 30 minutes, which was just enough for completing the echocardiography. This observation corresponds to results from Tamura *et al*. [10] in which dogs treated with 5 mg kg^-1^ of alfaxalone IM remained recumbent for 36 ± 28 minutes. Intravenous administration of 4 mg kg^-1^ alfaxalone produced anaesthesia for 25 ± 7 minutes in healthy Beagles [5]. The shorter duration of sedation may be explained by the lower, but more sustained, peak plasma concentrations generally achieved after IM administration in comparison with the IV route. In our study, only a few dogs reached the maximal sedation score possibly because the dose of alfaxalone was too low. Indeed, the neurodepression score appears to be dose-dependent [10]. The quality and depth of sedation is difficult to evaluate objectively. The comparison with other studies is also difficult because they use different sedation scores. In absence of a validated sedation score for dogs, a composite simple descriptive sedation score modified from Gurney *et al*. [20] was chosen to evaluate sedation in our patients. Taking into account posture, appearance, interactive behaviour, restrainability, response to noise and general attitude, this subjective score was made to be as discriminating as possible. We expanded the existing score to include the response to shoulder touch, as used in a sedation score by Smith *et al*. [26]. It should be noted that the sedation scores obtained after IM injection have a great individual variability. This is evident from the wide range of each data (Table 1 and Fig 3) and the large standard deviation observed in Tamura *et al*.’s study [10], which were not observed by Rodriguez *et al*.’s [5] following IV administration. Therefore, the precise duration of sedation based on scores is difficult to estimate.

In our study, times of sedation were similar to those observed by Tamura *et al*. [10] after alfaxalone 5 mg kg^-1^ IM (VFMT: 28 ± 10 minutes vs 26 ± 18 minutes; HLT: 31 ± 8 minutes vs 37 ± 28 minutes, DecT 42 ± 14 minutes vs 36 ± 28 minutes, respectively), except for UST (48 ± 12 minutes vs 84 ± 31 minutes, respectively) and RecT (16 ± 8 minutes vs 34 ± 8 minutes, respectively). The difference in UST might result from the definition of “unaided standing” including or not the period of post standing ataxia. The addition of medetomidine and butorphanol to 2.5 mg kg^-1^ alfaxalone IM doubled the sedation times [12]. Times of sedation (induction, maintenance, and recovery) are defined differently depending on the study. For example, some studies define the duration of sedation as the time between lateral recumbency and extubation while others define it as the time between lateral recumbency and first head lift, which may preclude the comparisons between studies. The duration of sedation defined as the time from induction to extubation was shorter after IV than IM administration (9.8 ± 2.4 minutes; 13.8 ± 4.6 minutes; 31.4 ± 6.9 minutes; 75.1 ± 28.9 minutes at 2, 3, 6 and 20 mg kg^-1^, respectively) [4,6].

Most studies report that the quality of recovery from alfaxalone anaesthesia alone in dogs is “quite smooth” to “excellent” [4,10,24,27]. However, it was also reported that dogs exhibited transient muscular tremor, staggering gait, paddling of the forelimb, muscular twitching, limb extension or vocalization during the early recovery phase [3,10]. Ferchichi *et al*. [28] suggested that the dogs were no longer exposed to alfaxalone during the recovery phase from a 50–90 minutes-anaesthesia produced by 2 mg kg^-1^ alfaxalone IV associated with methadone and that the excitement observed during recovery could not be considered as a side effect of alfaxalone. We also observed some of these events in our study and it was suggested that they are more common when alfaxalone is administered IM as compared with the IV route because of differences in pharmacokinetics [10]. In our study, the time needed to clear the plasma from the drug (considering five times the plasma terminal half-life) would be approximatively 2h30, which is longer than the duration of the sedation. Therefore, the dogs were most likely still exposed to alfaxalone during the recovery phase as observed in Fig 3. Nevertheless, in accordance with Ferré *et al*.’s [14] observations, the agitation occurred only in response to either manipulation or sounds during sedation. Further studies would be required to evaluate the effect of premedication with butorphanol on the quality of sedation and recovery from IM alfaxalone.

The present study had several limitations. First, the small sample size may have limited the power of the tests involving echocardiographic measurements. Second, the population studied was healthy young dogs. As a consequence, this study did not address the safety of administering alfaxalone IM to dogs with heart disease or old dogs (more representative of the population of dogs with heart disease). Furthermore, the short duration of sedation did not allow to study repeated echocardiographic measurements over time. The fact that animals underwent the experiment in the same order could have created a bias, and a learning curve or an adaptation of the animals to instrumentation, decreasing or increasing their stress levels, could not be excluded. For this reason, we compared the results of HR, MAP and *f*R between the first and second sedation. Although mean values for all parameters were lower during echocardiography than during sedation scoring, the trend was comparable. This suggested that a familiarisation of the animals to the experimental setting could have occurred thereby decreasing their stress levels before sedation, but could also have indicated that the drug’s action itself is responsible for the trend.

## Conclusions

Although the duration of sedation achieved with 4 mg kg^-1^ alfaxalone was sufficient and changes in the echocardiographic measurements remained limited, IM alfaxalone alone may not be considered an ideal sedative drug during echocardiography due to its significant influence on HR. Further studies would be required to evaluate the effect of alfaxalone in dogs with cardiac diseases and the impact of the association between IM alfaxalone with butorphanol on the quality of sedation for echocardiography in dogs
